# Effect of Pulse Plasma Sintering Temperature on Microstructure and Mechanical Properties of Al_2_O_3_-Cu Composites

**DOI:** 10.3390/ma19061086

**Published:** 2026-03-12

**Authors:** Paulina Piotrkiewicz, Justyna Zygmuntowicz, Marcin Wachowski, Ireneusz Szachogłuchowicz, Waldemar Kaszuwara

**Affiliations:** 1 Faculty of Materials Science and Engineering, Warsaw University of Technology, 141 Woloska St., 02-507 Warsaw, Poland; paulina.piotrkiewicz@gmail.com (P.P.); waldemar.kaszuwara@pw.edu.pl (W.K.); 2 Faculty of Mechanical Engineering, Military University of Technology, 2 Gen. S. Kaliskiego St., 00-908 Warsaw, Poland; marcin.wachowski@wat.edu.pl (M.W.); ireneusz.szachogluchowicz@wat.edu.pl (I.S.)

**Keywords:** Al_2_O_3_-Cu, pulse plasma sintering, PPS, ceramic-metal composites, microstructure, liquid phase

## Abstract

Al_2_O_3_-Cu ceramic-metal composites containing 2.5 vol.% of a metallic phase were fabricated using the Pulse Plasma Sintering (PPS) method in order to evaluate the influence of sintering temperature on densification, microstructure, and mechanical performance. Consolidation was carried out at 1200 °C, 1250 °C, 1300 °C, and 1400 °C under uniaxial pressure with a short sintering time of 3 min. Regardless of the processing temperature, all composites exhibited very high relative densities exceeding 99% of the theoretical value, indicating the high efficiency of PPS in densifying Al_2_O_3_-Cu systems while suppressing copper leakage. X-ray diffraction confirmed the presence of only two phases, Al_2_O_3_ and Cu, with no secondary reaction products. Microstructural observations revealed irregular copper particles and areas of dispersed metallic phase, whose proportion decreased with increasing sintering temperature due to accelerated matrix densification and copper immobilization. Grain growth in the alumina matrix was strongly temperature-dependent, with the average equivalent grain diameter increasing from 0.49 µm at 1200 °C to 2.35 µm at 1400 °C. Hardness decreased from 19.5 ± 2.8 GPa to 12.2 ± 1.6 GPa with increasing temperature, whereas fracture toughness reached a maximum of 5.42 ± 0.65 MPa·m0.5 at 1400 °C. The highest strength under monotonic compression conditions was obtained for samples sintered at 1300 °C, indicating an optimal balance between densification and microstructural coarsening. These results demonstrate that PPS is an effective method for producing dense Al_2_O_3_-Cu composites with tailored microstructure and mechanical properties.

## 1. Introduction

Alumina-based ceramic-metal composites are an important class of advanced structural and functional materials [1,2,3]. They combine the hardness, thermal stability, and wear resistance of ceramics with the electrical and thermal conductivity and damage tolerance of metallic phases [4,5,6,7]. Al_2_O_3_-Cu composites are of particular interest in this context due to copper’s exceptionally high electrical and thermal conductivity, which enables the tailoring of multifunctional properties while preserving the intrinsic advantages of an alumina matrix [8,9]. These materials are ideal for use in thermal management, electrical components, and mechanically loaded systems operating under challenging conditions.

Despite these advantages, fabricating Al_2_O_3_-Cu composites remains a significant technological challenge. The main difficulty is the significant differences in physicochemical properties between alumina and copper, including melting point, thermal expansion coefficient, and interfacial wettability. Copper melts at 1083 °C [10], which is well below the conventional sintering temperature of over 1500 °C required for effective densification of Al_2_O_3_ [11,12,13,14]. Consequently, conventional pressureless sintering often results in uncontrolled copper migration, metal leakage, residual porosity, and microstructural heterogeneity, all of which compromise the mechanical and functional performance of the composite. Various approaches have been proposed to mitigate these issues, including hot pressing [15,16,17], spark plasma sintering [18,19], and atmosphere-controlled sintering [20,21,22]. However, achieving near-theoretical density while maintaining a stable and well-distributed copper phase remains challenging.

In this context, pulse plasma sintering (PPS) emerges as a promising alternative consolidation technique for ceramic-metal composites [23,24]. In recent years, pulse plasma sintering (PPS) has emerged as a distinct current-assisted consolidation technique for advanced ceramic and ceramic-metal systems [25]. In contrast to conventional pressureless sintering and hot pressing, PPS employs high-energy electrical pulses generated by capacitor discharge, resulting in extremely high heating rates, very short dwell times, and simultaneous application of uniaxial pressure [26,27,28]. Unlike spark plasma sintering (SPS), where a quasi-continuous pulsed DC is applied, PPS is characterized by discrete, high-intensity current impulses with short duration, which promote rapid Joule heating of the conductive tooling and compaction [29]. These conditions enable accelerated neck formation, enhanced mass transport, and efficient densification at reduced effective thermal exposure.

Previous studies have demonstrated that PPS can successfully densify oxide ceramics and ceramic-metal composites while limiting excessive grain growth and suppressing deleterious liquid-phase migration phenomena [30,31,32]. In alumina-based systems, PPS has been shown to achieve near-theoretical density within minutes, even in compositions that are challenging to consolidate by conventional routes. Importantly, in composite systems containing low-melting metallic phases, the ultrafast densification kinetics associated with PPS may promote early formation of a mechanically rigid ceramic skeleton, thereby physically immobilizing the molten metal and reducing leakage or macroscopic segregation [30,31,32].

Despite these advantages, the temperature-dependent interplay among matrix densification, grain growth, and liquid-metal redistribution in binary Al_2_O_3_-Cu systems processed by PPS has not yet been systematically elucidated. Most prior studies on alumina–copper composites have focused either on alternative processing routes, such as atmosphere-controlled sintering and SPS, or on higher metal fractions where percolation and infiltration mechanisms dominate. In contrast, the present work deliberately investigates a low copper content (2.5 vol.%) below the percolation threshold to isolate the fundamental immobilization mechanisms of a discontinuous metallic phase under ultrafast PPS conditions.

By systematically varying the sintering temperature between 1200 °C and 1400 °C while maintaining identical pressure and dwell time, this study establishes direct correlations between processing temperature, alumina grain growth, copper redistribution, and mechanical response. Particular emphasis is placed on identifying the temperature window where efficient copper retention, controlled microstructural coarsening, and an optimal strength-toughness balance coexist. In this way, the work advances beyond previous PPS studies by providing a temperature-resolved structure-property framework for a binary Al_2_O_3_-Cu composite system with low metallic phase content.

The application of PPS offers several potential advantages for Al_2_O_3_-Cu composites. The rapid sintering kinetics promote the early bonding of alumina particles, which can mechanically immobilise the molten copper and prevent its uncontrolled flow or loss from the compact [24,33]. At the same time, the applied pressure enables the metallic phase to fill intergranular voids, thereby contributing to pore elimination and high densification. However, the interplay between processing temperature, copper redistribution, matrix grain growth, and the resulting mechanical behaviour in PPS-processed Al_2_O_3_-Cu composites has not yet been fully clarified.

The objective of this study is therefore to investigate the formation, microstructure, and selected physical and mechanical properties of Al_2_O_3_-Cu composites containing 2.5 vol.% of a metallic phase. These composites were fabricated using the Pulse Plasma Sintering method. Composite samples were produced at various sintering temperatures (1200–1400 °C), enabling the impact of processing temperature on densification, phase stability, copper distribution, grain growth, hardness, fracture resistance, and compressive strength to be evaluated. Particular focus is given to the mechanisms governing copper immobilisation within the alumina matrix, the evolution of microstructural features under PPS conditions, and the resulting structure-property relationships.

The results presented in this study shed new light on the consolidation behaviour of Al_2_O_3_-Cu composites under pulsed plasma conditions, demonstrating the potential of PPS as an effective processing method for ceramic-metal systems containing low-melting metallic phases. These findings further our understanding of advanced sintering techniques and support the development of dense, defect-free alumina-based composites with customisable mechanical and functional properties.

## 2. Materials and Methods

A commercially available α-Al_2_O_3_ ceramic powder (TM-DAR, Taimei Chemicals, Japan) was employed as the ceramic matrix material. The metallic component consisted of copper powder supplied by Sigma-Aldrich (Sigma-Aldrich, St. Louis, MO, USA), which exhibited a dendritic particle morphology with branches of non-uniform size. The copper powder had a particle size below 150 µm (as specified by the manufacturer). The choice of copper powder as the basic metallic component was driven by its high plasticity, thermal conductivity (401 W/m·K [34]), and electrical conductivity [34]. The addition of copper to the ceramic matrix, in addition to increasing resistance to brittle fracture, enables control of properties such as thermal and electrical conductivity, which, in the case of copper, are significantly higher than those of pure corundum ceramics.

The manufacturing process was carried out using the Pulse Plasma Sintering (PPS) method. This method is successfully used to produce ceramic-metal composites, including composites with copper as the reinforcing phase. Literature data show that the selected method for forming shapes enables the production of high-density materials [35,36,37]. As part of this work, ceramic-metal composites were produced with a metal phase content of 2.5% by volume. All metals used in the process were in powder form. In all the samples produced, the ratio of metal powders used was 1:1. The composite samples were formed by plasma-spark sintering at four different temperatures: 1200 °C, 1250 °C, 1300 °C, and 1400 °C. The melting point of copper (1083 °C [10,34]) is lower than the standard sintering temperature for Al_2_O_3_-based materials. The possibility of lowering the sintering temperature is therefore one potential solution to reduce liquid copper losses during consolidation. The first stage of forming involves placing the prepared mixture of Al_2_O_3_-Cu powders in a graphite mold lined with graphite foil between two stamps wrapped in a carbon mat.

The graphite foil increases the contact area, improving current flow through the sample during the process. The carbon mat is used to reduce heat loss. The process was carried out in a high vacuum environment at a pressure of 10^−6^ mbar. Capacitor banks were used as the current source. The pulse was generated on an ignitron. A short circuit occurred when the current pulse jumped between the transmitting and receiving electrodes, causing current to flow through the conductive graphite matrix. Graphite stamps acted as electrodes. As a result of the current flow, the matrix, which was the heat source, heated due to Joule heating.

The heating process was controlled by varying the voltage and current. The heating rate was 220 °C/min. Four sintering temperatures were used: 1200 °C, 1250 °C, 1300 °C, and 1400 °C. The sintering time was 3 min in each case. The simultaneous forming and sintering of the sample was carried out in two stages: first, applying a pre-pressure of 20 MPa to give the sample the appropriate geometry. The formed shape was then compacted at 80 MPa. The sintering process conditions were selected based on previous research to enable sintering of the ceramic matrix [24]. The samples produced via PPS had a diameter of 22 ± 2 mm and a height of 5 ± 2 mm. For each tested temperature, 10 samples were produced and tested.

The selection of a copper content of 2.5 vol.% is scientifically justified and appropriate for the objectives of the present study. At this low metallic phase fraction, the composite remains well below the percolation threshold for copper in an alumina matrix. In ceramic–metal systems with randomly distributed metallic particles, electrical and thermal percolation typically occurs when the metal content exceeds 15–20 vol.%, depending on particle morphology and connectivity. At 2.5 vol.% Cu, the metallic phase is therefore discontinuous and isolated and does not form an interconnected network within the ceramic matrix. This ensures that composite behaviour is dominated by the alumina skeleton, with copper acting as a discrete secondary phase rather than a load-bearing or transport controlling framework. Importantly, keeping the copper content below the percolation threshold enables the study to concentrate on fundamental consolidation and interfacial phenomena rather than collective metallic effects. In particular, it eliminates complications associated with continuous liquid-metal flow, capillary-driven infiltration, or electrically assisted percolation pathways, which would obscure the interpretation of densification mechanisms under pulse plasma sintering conditions. Consequently, any observed suppression of copper leakage, high densification efficiency, or microstructural stabilisation can primarily be attributed to the rapid formation of a mechanically rigid alumina skeleton and the physical immobilisation of the molten copper phase rather than percolation-driven confinement.

Furthermore, using 2.5 vol.% Cu effectively creates a model composite system for isolating copper immobilisation mechanisms during PPS. At this low concentration, there is still sufficient copper to interact with the evolving alumina grain boundaries and intergranular regions, without dominating the sintering kinetics or altering the intrinsic grain-growth behaviour of the ceramic matrix. This enables the direct examination of how PPS-specific features, such as high heating rates, short dwell times, and applied uniaxial pressure, govern the retention, redistribution, and stabilisation of a low-melting metallic phase within a rapidly densifying ceramic framework.

In this context, the chosen copper content strikes a deliberate balance between experimental sensitivity and mechanistic clarity. Higher metal fractions would promote extensive liquid-phase effects, agglomeration, and potential percolation, complicating interpretation and masking the role of early-stage ceramic bonding. Conversely, significantly lower metal contents could limit the statistical relevance of metallic-phase interactions. Therefore, a composition of 2.5 vol.% Cu is optimal for investigating the fundamental mechanisms of liquid-metal immobilisation and microstructural control in Al_2_O_3_-Cu composites processed by PPS, while remaining relevant to the design of practical ceramic–metal composites.

The fundamental physical characteristics of the sintered composites were evaluated using the Archimedes immersion technique. Measurements were conducted in accordance with the requirements of PN-EN ISO 18754:2022-10 [38]. The theoretical density of the Al_2_O_3_-Cu composite was determined using the rule of mixtures, based on the known densities of the constituent phases and their nominal volume fractions (2.5 vol.% Cu and 97.5 vol.% Al_2_O_3_). The reference densities used were 3.98 g/cm^3^ for Al_2_O_3_ and 8.96 g/cm^3^ for Cu. The relative density was subsequently determined using the Archimedes method (immersion in distilled water), following standard practice for dense ceramic materials. Each measurement was repeated 5 times to ensure reproducibility, and the average values are reported. Firstly, the dry mass of each specimen was determined in air. The samples were then immersed in demineralised water until saturated, after which their mass was measured in air and while suspended in the liquid. These mass values were then used to calculate the relative density, water absorption, and open porosity of the sintered materials. For each series, 10 samples were tested.

Phase constitution of the sintered composites was examined by X-ray diffraction (XRD). The measurements were performed using a Rigaku Miniflex II powder diffractometer (Rigaku Corporation, Tokyo, Japan) equipped with a Cu Kα radiation source (λ = 1.54059 Å), operating at 30 kV and 15 mA. Diffraction patterns were collected over a 2θ range from 20° to 100°, with an angular step size of 0.01° and a counting time of 1 s per step. Phase identification was carried out by comparing the experimental patterns with reference data from the PDF+4 2022 database using the Jade 8.5 software package (Materials Data). The XRD study was conducted on a single sample at specific temperatures.

A preliminary assessment of the surface morphology and metallic phase distribution within the composite structure was conducted using an Olympus LEXT OLS4100 confocal laser scanning microscope (Olympus Corporation, Tokyo, Japan). This analysis provided qualitative information on the spatial arrangement of the metallic component at the surface of the sintered samples.

Detailed microstructural observations of both polished surfaces and fracture cross-sections were performed using a JEOL JSM-6610 scanning electron microscope (SEM) (JEOL Ltd., Tokyo, Japan). Imaging was carried out in secondary electron (SE) and backscattered electron (BSE) modes at an accelerating voltage of 15 kV. Prior to examination, all specimens were coated with a thin conductive carbon layer using a JEOL Fine Coat Ion Sputter JFC-1100 (JEOL Ltd., Tokyo, Japan) to prevent surface charging.

The elemental composition and phase distribution were investigated further using energy-dispersive X-ray spectroscopy (EDS) with the SEM-EDS system integrated into the JEOL JSM-6610 microscope. Elemental maps were acquired for selected regions of interest, paying particular attention to the zones where the ceramic matrix meets the metallic phase.

Vickers hardness measurements were performed on polished, parallel surfaces of the sintered samples after standard metallographic preparation. Testing was performed using an HVS-30T hardness tester (Huayin Testing Instrument Co., Ltd., Laizhou, China). A constant load of 98 N was applied for 15 s in all measurements to ensure consistency across the sample series.

The resistance of the Al_2_O_3_-Cu composites to brittle fracture was assessed using an indentation fracture method. Fracture resistance was estimated based on Lankford’s relationship [39], which is applicable to various crack geometries. Crack lengths were measured for radial cracks emanating from the corners of Vickers indentations produced under a load of 98 N. Ten impressions were made for each sample tested at different temperatures.

Fracture toughness values were estimated using the indentation fracture method based on radial median crack lengths generated under Vickers loading. It should be emphasized that classical indentation fracture equations were originally developed for homogeneous, linear-elastic brittle materials and are strictly valid within the assumptions of linear elastic fracture mechanics. In multiphase ceramic-metal composites such as the present Al_2_O_3_-Cu system, these assumptions are only approximately satisfied. The incorporation of a ductile copper phase may introduce localized plastic deformation beneath the indenter, stress redistribution around the crack tip, crack bridging, and crack-tip shielding effects. Such mechanisms can modify crack-propagation behavior and may lead to deviations between indentation-derived values and the intrinsic fracture toughness determined using standardized fracture mechanics methods (e.g., SENB or Chevron-notch techniques). Consequently, the calculated K_IC_ values should be interpreted as apparent indentation fracture resistance rather than absolute material constants. Despite these limitations, several experimental observations justify the use of the IF method for comparative analysis within the investigated composite series. Radial-median crack systems were clearly observed for all compositions under identical indentation conditions, and the crack morphology remained consistent across samples. No extensive metal extrusion, excessive pile-up, or indentation-induced macroscopic ductile flow was detected at the applied loads, indicating that the alumina matrix remained the dominant load-bearing phase.

Furthermore, identical surface preparation procedures, indentation loads, and crack measurement protocols were used for all materials, ensuring methodological consistency. Therefore, although the absolute numerical values of K_IC_ should be interpreted with caution, the indentation method provides a reliable comparative assessment of relative crack propagation resistance within the Al_2_O_3_-Cu composite system. The observed trends in apparent fracture toughness are consistent with the materials’ microstructural features and mechanical performance, supporting the validity of the comparative analysis presented in this study.

The mechanical performance of the sintered composites was also evaluated through monotonic compression testing. The primary objective of this investigation was to quantify the compressive strength of the specimens and to analyse failure mechanisms using full-field strain measurements obtained via digital image correlation (DIC). Compression tests were performed using an Instron 8802 MT (Instron, Norwood, MA, USA) servo-hydraulic testing system equipped with dedicated control and acquisition software enabling continuous monitoring of the applied load and crosshead displacement. The specimens were positioned between the platens of a compression fixture installed in the testing machine grips. The compressive load was applied progressively until catastrophic failure occurred. Failure was defined as the point at which a distinct fracture event resulted in a sudden drop in the recorded load-displacement curve. This event was confirmed simultaneously by high-speed optical recording. The complete displacement response was continuously acquired throughout the test. Full-field strain evolution and deformation behaviour were monitored using a Dantec Q400 Digital Image Correlation system (Dantec Dynamics A/S, Skovlunde, Denmark). The system comprised two 8-megapixel cameras, providing a displacement measurement accuracy of ±0.01 pixels and a strain resolution of 0.01%. Data acquisition and post-processing were carried out using integrated ISTRA 4D software modules, which enabled visualization of strain maps and advanced quantitative analysis. The applied monotonic compressive loading allowed the determination of force-displacement characteristics for each specimen. All experiments were conducted under controlled laboratory conditions at an ambient temperature of 22 ± 2 °C. To obtain reliable results, 8 samples from each series were tested; the following section presents averaged force-displacement graphs and representative DIC images for each series.

Grain size analysis of the Al_2_O_3_ ceramic matrix was performed using stereological methods. Fracture surfaces were examined by scanning electron microscopy, and Al_2_O_3_ grains were manually identified with consideration of their three-dimensional morphology. Image processing procedures were subsequently applied to extract grain boundaries and generate binary images. Quantitative analysis was conducted using Micrometer software version 1.1 [40,41,42], which was used to calculate the average equivalent diameter (d_2_) for each sample. The parameter d_2_ represents the diameter of a circle with an area equivalent to that of the analyzed non-spherical grain [40]. Based on the obtained data, percentage distributions of the equivalent grain diameter were constructed for all investigated composites. To ensure statistically significant results, SEM micrographs of fracture surfaces were randomly selected, and a minimum of 1400 individual grains were counted for each composite at different temperatures.

## 3. Results and Discussion

**Figure 1:**
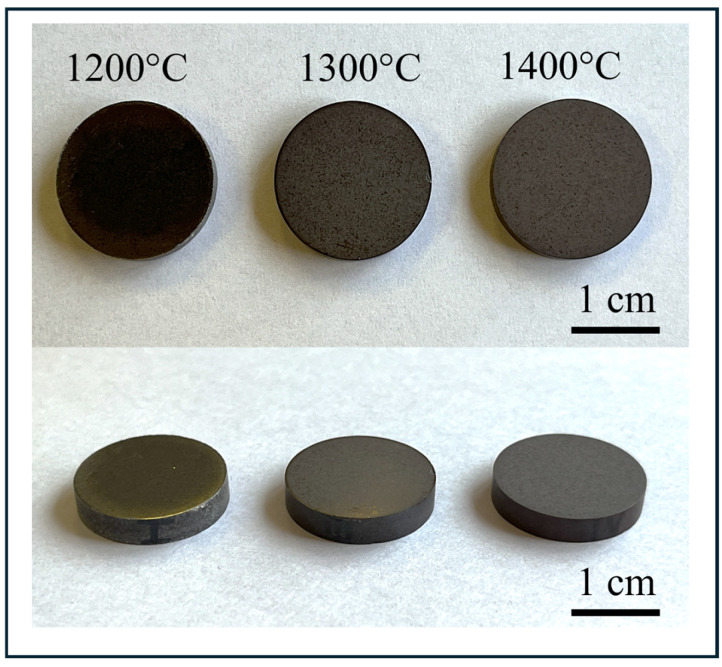
Macroscopic appearance of Al_2_O_3_-Cu composites (2.5 vol.% Cu) fabricated by Pulse Plasma Sintering (PPS) at 1200 °C, 1300 °C, and 1400 °C under 80 MPa and 3 min dwell time.

Sample photos of Al_2_O_3_-Cu composite samples with 2.5% vol. metal phase content obtained in this experiment are shown in Figure 1. Macroscopic observations of the manufactured samples revealed no surface defects for fittings manufactured using the PPS method. No copper leakage from the sample was observed during the process.

Analysis of the values obtained for Al_2_O_3_-Cu samples produced by this method shows no clear correlation between sintering temperature and relative density. All series produced were characterized by a very high level of compaction, exceeding 99% of the theoretical density (Table 1). The observed differences in compaction values between samples produced by different methods are probably related to the use of compaction pressure during sintering in the PPS forming process. Copper, which is in a liquid state during the process, can, under applied pressure, fill the spaces between Al_2_O_3_ matrix grains before they bond due to sintering. This contributes to the elimination of internal pores and, consequently, to the observed high relative density of the obtained samples, regardless of the process temperature. At the same time, the proportion of copper in the sample volume is so small that it is retained during sintering by the connecting Al_2_O_3_ grains. Hence, no visible macroscopic defects were observed after the process. In uniaxial pressing with free sintering, no additional pressure is applied to aid copper migration or eliminate pores, especially at lower sintering temperatures. The consequence may be a clear dependence of the obtained density on the sintering temperature, as seen in these samples. Although the overall bulk density approaches theoretical values, localized microstructural features such as residual closed porosity, copper phase redistribution, ceramic–metal interfacial characteristics, and potential thermal residual stresses may still act as strength-limiting defects.

**Figure 2:**
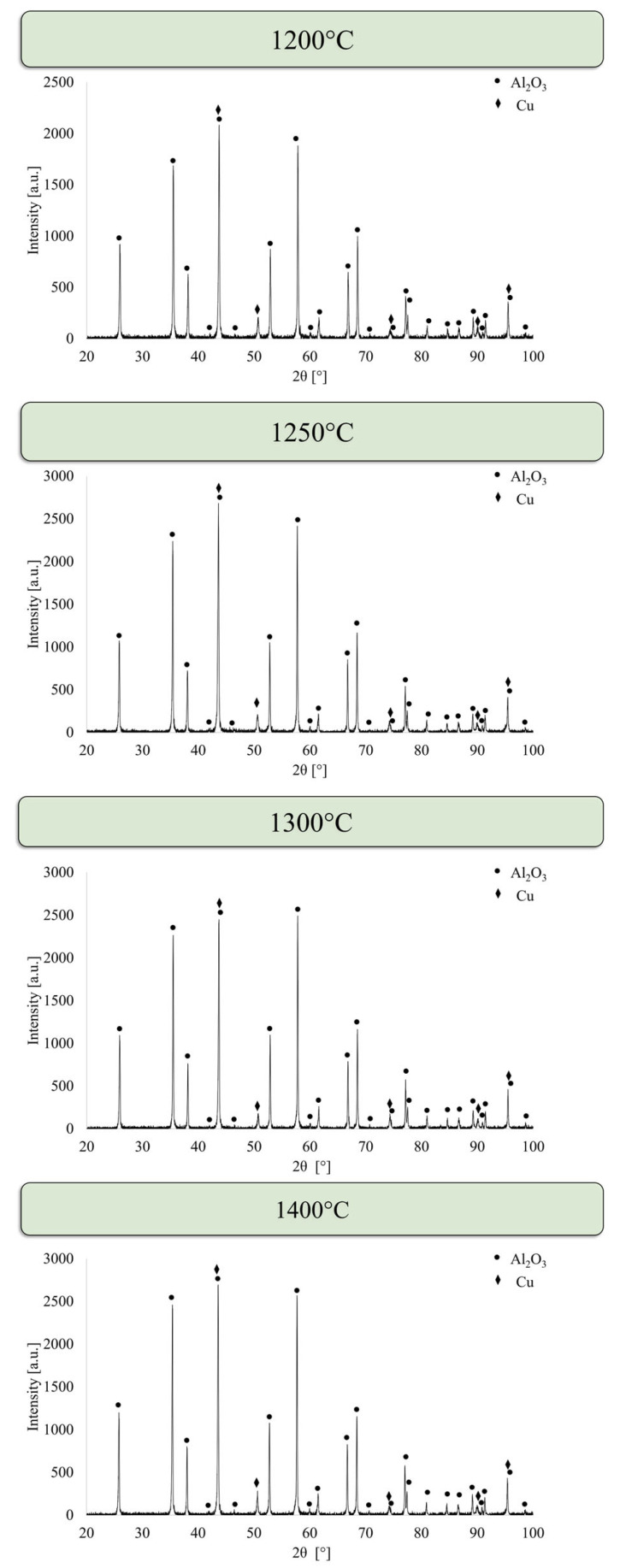
X-ray diffraction (XRD) patterns of Al_2_O_3_-Cu composites (2.5 vol.% Cu) sintered by PPS at 1200 °C, 1250 °C, 1300 °C, and 1400 °C. Diffraction peaks correspond exclusively to corundum-structured Al_2_O_3_ (PDF #98-000-0174) and metallic Cu (PDF #04-003-5633).

Figure 2 shows the results of phase-composition analysis of samples from Al_2_O_3_-Cu systems containing 2.5% metallic phase by volume. The phase analysis results showed the presence of two phases: Al_2_O_3_ with a corundum structure (PDF #98-000-0174) and Cu (PDF #04-003-5633). No effect of the process temperature on the phase composition of the samples was observed.

**Figure 3:**
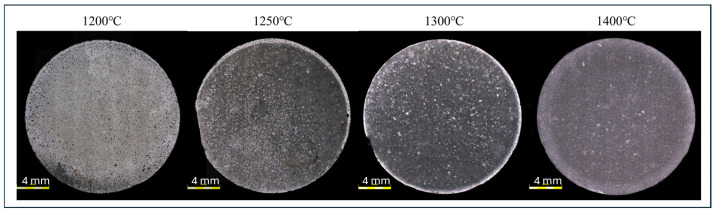
Confocal laser scanning microscopy images of the polished surfaces of Al_2_O_3_-Cu composites (2.5 vol.% Cu) sintered by PPS at 1200 °C, 1250 °C, 1300 °C, and 1400 °C.

Figure 3 shows the results of observations of the surface of composite samples with 2.5% vol. of the metallic phase formed by plasma-pulse sintering. The influence of the process temperature on the surface characteristics obtained was observed. It was found that, despite high compaction, metal phase losses are visible in fittings formed at temperatures ranging from 1200 °C to 1300 °C, with the amount decreasing with increasing process temperature. In addition, it was observed that copper particles merged into larger clusters during the forming process. The observed sample surfaces are similar in character to those of freely sintered fittings. The PPS sample surface formed at 1400 °C showed a significantly lower proportion of large metallic particles. No areas depleted in the metallic phase or defects resulting from possible metal flow from the sample during forming were observed in the material. The distribution of the metallic phase in the matrix was more homogeneous than in the other samples for this system.

**Figure 4:**
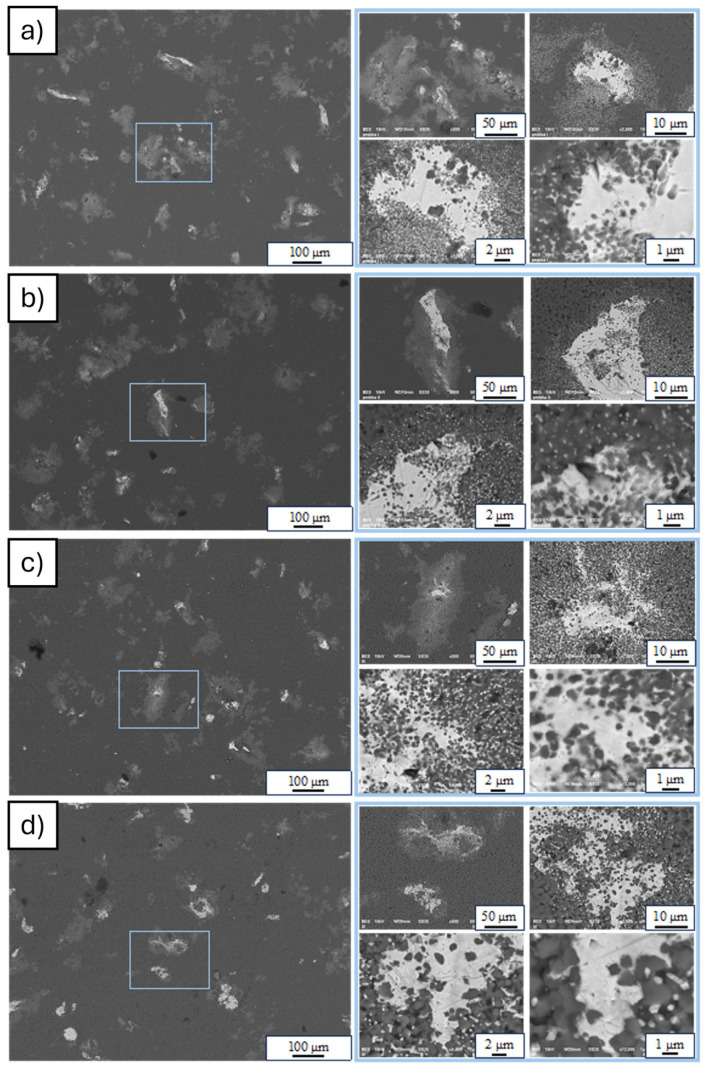
Scanning Electron Microscopy (SEM) micrographs (secondary electron mode) of polished cross-sections of Al_2_O_3_-Cu composites (2.5 vol.% Cu) fabricated by PPS at: (**a**) 1200 °C, (**b**) 1250 °C, (**c**) 1300 °C, (**d**) 1400 °C.

Next, SEM micrograph analysis revealed that Al_2_O_3_-Cu samples containing 2.5% vol. of the metallic phase were produced using the PPS method (Figure 4). Microscopic examination revealed irregularly shaped metallic particles in the tested samples. The solid Cu particles were surrounded by regions containing a mixture of matrix particles and isolated metal particles. The observed microstructure was probably due to additional compaction pressure acting on the material during sintering. Under pressure, copper migrates, filling the free spaces between the matrix particles. Due to the lack of wettability between the matrix and the metallic phase, its expansion is inhibited only as a result of physical immobilization between the sintered Al_2_O_3_ particles. In the case of the pulsed plasma sintering process, Al_2_O_3_ particles sinter much faster than in the conventional free sintering process. For this reason, in the case of PPS formed shapes, the copper particles were closed more quickly by the matrix particles, which in turn made it possible to obtain a microstructure characterized by the absence of large agglomerates of metal particles.

**Figure 5:**
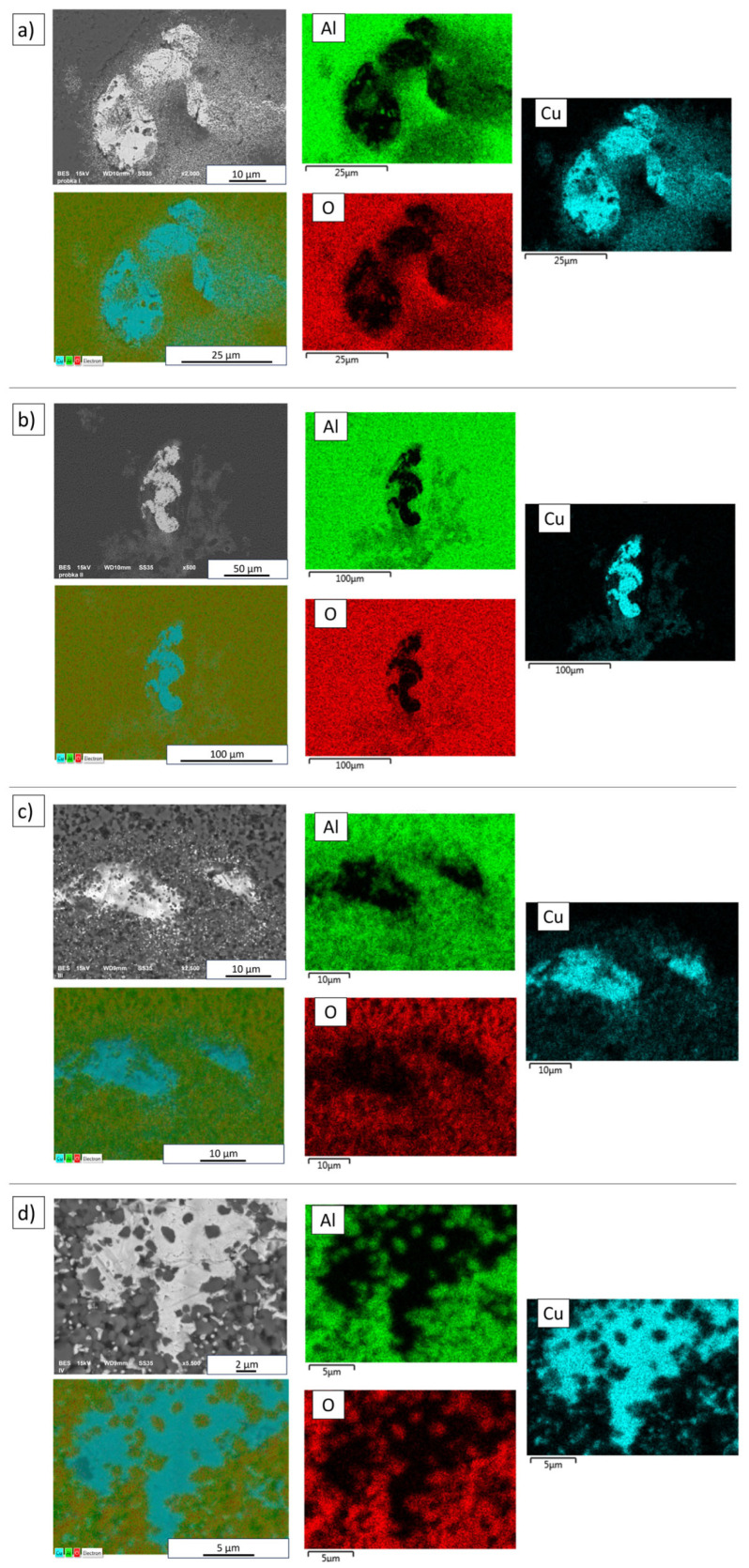
SEM-EDS elemental mapping of Al_2_O_3_-Cu composites (2.5 vol.% Cu) sintered by PPS at: (**a**) 1200 °C, (**b**) 1250 °C, (**c**) 1300 °C, (**d**) 1400 °C.

Analysis of X-ray microanalysis results for the chemical composition of samples produced using the PPS method revealed signals from three elements: Al, O, and Cu (Figure 5). The study confirmed the presence of copper in both solid metal particles and observed areas of dispersed metal phase. Aluminum and oxygen were present both in the microstructure of the matrix and in areas with a dispersed metallic phase. During sintering, copper filled the spaces between the matrix particles and was then enclosed by them as the process progressed, as confirmed by the results. Based on the analysis, no differences related to the temperature used in the process were found.

Hardness tests revealed that hardness decreased with increasing process temperature. The highest hardness was observed in the series produced at 1200 °C (19.5 ± 2.8 GPa). The average hardness of samples sintered at 1250 °C was 18.1 ± 1.3 GPa, while for samples produced at 1300 °C it was 13.3 ± 2.0 GPa. The tests showed that the Al_2_O_3_-Cu sample produced by the PPS method at 1400 °C had the lowest hardness. The average hardness value in this case was 12.2 ± 1.6 GPa. The systematic decrease in Vickers hardness with increasing sintering temperature can be partially explained by a Hall-Petch relationship, which relates hardness or strength to the inverse square root of grain size [43,44,45,46]. For alumina-based ceramics, numerous studies have reported that grain boundary strengthening plays a significant role in determining hardness, particularly in fine-grained and submicron microstructures, where grain boundaries effectively impede dislocation motion and crack initiation [47,48,49,50,51]. In the present Al_2_O_3_-Cu composites, the average equivalent alumina grain diameter increased from approximately 0.49 µm at 1200 °C to 2.35 µm at 1400 °C, which corresponds well with the monotonic reduction in hardness from 19.5 ± 2.8 GPa to 12.2 ± 1.6 GPa. This qualitative trend is consistent with Hall-Petch behavior, in which increasing grain size reduces resistance to localized deformation beneath the indenter.

However, a strictly quantitative Hall-Petch relationship cannot be assumed for the investigated composites due to their multiphase nature and the presence of a ductile metallic phase. Unlike monolithic alumina, where hardness is primarily governed by grain size and grain boundary density, the Al_2_O_3_-Cu system exhibits additional deformation mechanisms associated with the copper phase. Localized plastic deformation of copper, stress redistribution at ceramic-metal interfaces, and crack-metal interactions beneath the Vickers indenter may all contribute to the measured hardness values, particularly at higher sintering temperatures, when copper is more homogeneously distributed, and alumina grains are coarser. Moreover, the broadening of the grain size distribution at elevated temperatures further complicates the direct application of a Hall-Petch-type model. In such cases, hardness reflects an averaged response of regions with different local grain sizes and varying proximity to metallic inclusions, rather than a single characteristic microstructural length scale. As a result, deviations from ideal Hall-Petch behavior are expected, especially in the coarser-grained regime where the influence of grain boundaries on deformation becomes less dominant. Therefore, while the observed decrease in hardness with increasing sintering temperature is qualitatively consistent with Hall-Petch-type grain-size strengthening of the alumina matrix, the measured values should be interpreted as the outcome of coupled grain growth and composite-specific effects rather than a direct manifestation of a classical Hall-Petch relationship. The results highlight that in alumina-based ceramic–metal composites, hardness is controlled by a combination of matrix grain size, metallic phase distribution, and interfacial interactions, all of which evolve with PPS processing temperature.

The fracture toughness values reported in this study were estimated using the indentation fracture method, which is widely applied for comparative assessment of brittle and quasi-brittle ceramic materials [52,53]. Nevertheless, it is important to explicitly acknowledge the inherent limitations of this approach, particularly when applied to heterogeneous ceramic-metal composites such as the Al_2_O_3_-Cu system investigated here. The method is sensitive to assumptions about crack geometry and crack-indent interactions, and deviations from ideal radial median crack patterns can introduce uncertainty into the calculated K_IC_ values. In multiphase materials, additional complexity arises from local microstructural heterogeneities that may influence crack initiation and propagation beneath the indenter. In the present composites, the presence of a ductile copper phase may locally affect the stress field generated during indentation. Plastic deformation of copper in the vicinity of the indent can partially relax stresses and alter crack lengths, potentially leading to either overestimation or underestimation of fracture toughness depending on the local distribution of the metallic phase. Moreover, the applicability of Lankford’s relationship, originally developed for monolithic, relatively homogeneous ceramics, to ceramic-metal composites should be approached with caution, as the underlying assumptions do not explicitly account for metallic-phase plasticity or interfacial effects [39]. Despite these limitations, the indentation fracture method was selected due to its suitability for systematic, comparative analysis across a series of samples processed under different sintering conditions, where specimen geometry and size constraints preclude the use of standardized macroscopic fracture toughness tests. To mitigate uncertainties, fracture toughness measurements were performed using multiple indentations on each sample, and the reported values represent statistically averaged results with corresponding standard deviations. Care was also taken to measure only well-developed radial cracks and to exclude indents exhibiting irregular or asymmetric crack patterns. Accordingly, the absolute K_IC_ values reported in this work should be interpreted as approximate indicators of fracture resistance rather than exact material constants. However, because all measurements were conducted under identical testing conditions and analyzed using the same methodology, the observed trends in fracture toughness as a function of sintering temperature remain meaningful and reliable. In particular, the systematic increase in K_IC_ at higher PPS processing temperatures reflects genuine changes in microstructure and crack microstructure interactions, even if the precise numerical values are subject to method-related uncertainty.

For Al_2_O_3_-Cu samples, the K_IC_ value for the shape produced at 1400 °C was 5.42 ± 0.65 MPa·m^0.5^. These samples had the highest resistance to brittle fracture among the samples produced. The lowest K_IC_ value was obtained for samples produced by the PPS method at 1200 °C, with a value of 4.38 ± 0.51 MPa·m^0.5^. The resistance to brittle fracture for samples produced at 1250 °C was 4.72 ± 0.91 MPa·m^0.5^, while for samples formed at 1300 °C it was 4.41 ± 0.47 MPa·m^0.5^.

The non-monotonic dependence of compressive strength on sintering temperature, with a maximum observed at 1300 °C, reflects a balance between competing microstructural mechanisms rather than a direct correlation with either grain size or fracture toughness alone. At temperatures up to 1300 °C, increasing the sintering temperature promotes more complete densification of the alumina matrix, improved interparticle bonding, and a reduction in critical microstructural defects, such as residual pores and weak grain boundaries. These effects enhance the load-bearing capacity of the ceramic skeleton and delay the onset of macroscopic cracking under compressive loading, resulting in a substantial increase in compressive strength compared with samples sintered at lower temperatures. Beyond 1300 °C, increasing the sintering temperature further leads to pronounced grain growth in the Al_2_O_3_ matrix and progressive microstructural coarsening. Although densification remains high, the growth and increasing heterogeneity of alumina grains reduce the density of grain boundaries that act as effective barriers to crack initiation. Larger grains are also more susceptible to the formation of critical flaws at grain boundaries and triple junctions, which can serve as preferential crack nucleation sites under compressive stress. Consequently, the intrinsic strength of the ceramic matrix decreases, even as fracture toughness continues to increase due to enhanced crack deflection and energy-dissipating mechanisms. At 1400 °C, the more homogeneous distribution and stronger mechanical constraint of the copper phase contribute to fracture toughness by promoting crack bridging and localized plastic deformation. However, these mechanisms primarily affect crack propagation rather than crack initiation. Compressive strength, particularly as measured by the Brazilian test, is governed predominantly by the stress required to initiate catastrophic fracture in the ceramic matrix [54,55]. Once a critical crack forms, the increased toughness is insufficient to compensate for the reduced matrix strength associated with grain coarsening, resulting in a lower peak compressive strength compared to the 1300 °C samples.

To conclude, the peak in compressive strength at 1300 °C corresponds to an optimal microstructural state in which high densification, fine-to-moderate alumina grain size, and effective copper immobilization coexist. At higher temperatures, the beneficial effects of improved interfacial interactions and metallic-phase toughening are outweighed by the deleterious impact of excessive grain growth on matrix strength. This divergence between strength and toughness highlights the importance of identifying property-specific processing windows in Al_2_O_3_-Cu composites fabricated by PPS and underscores that maximum toughness does not necessarily coincide with maximum strength.

The increase in fracture toughness observed for composites sintered at higher temperatures, particularly for samples processed at 1400 °C, cannot be attributed solely to alumina grain growth and should be considered as the result of multiple, interacting microstructural factors. While grain coarsening of the Al_2_O_3_ matrix is known to influence crack propagation paths, often promoting increased crack deflection and tortuosity, the magnitude of the toughness enhancement observed in the present study suggests that additional mechanisms contribute to the resistance against brittle fracture. One important factor is the evolution of interfacial cohesion between the copper phase and the alumina matrix with increasing sintering temperature. Microstructural observations indicate a more homogeneous distribution of copper and a reduced fraction of finely dispersed metallic regions at higher temperatures, which implies more effective immobilization of the metallic phase within the ceramic skeleton. Such microstructural stabilization is consistent with improved mechanical interlocking at the metal-ceramic interface, which can enhance load transfer and delay interfacial debonding during crack propagation. Stronger interfacial cohesion increases the likelihood of crack deflection along the interface or crack bridging by the metallic phase, both of which are recognized toughening mechanisms in ceramic-metal composites.

Furthermore, higher sintering temperatures promote more complete alumina-alumina bonding at grain boundaries, which may indirectly improve the integrity of the metal-ceramic interfaces by more effectively constraining copper particles. Under these conditions, the metallic phase is more likely to undergo localized plastic deformation when intersected by a propagating crack, dissipating fracture energy and thereby increasing apparent toughness. This interpretation is supported by the higher displacement at failure observed in compression tests for samples sintered at 1300 °C and 1400 °C, indicating an increased capacity for energy absorption prior to catastrophic fracture. Nevertheless, it must be emphasized that the indentation fracture method used to estimate fracture toughness does not allow for a direct, quantitative separation of grain size effects from interfacial contributions. The observed increase in K_IC_ therefore reflects a combined response of grain growth, copper redistribution, and potential enhancement of interfacial cohesion rather than a single dominant mechanism. The absence of detectable reaction phases at the Al_2_O_3_-Cu interface further suggests that any improvement in interfacial cohesion is likely mechanical in nature, arising from enhanced physical constraint and contact quality rather than chemical bonding.

**Figure 6:**
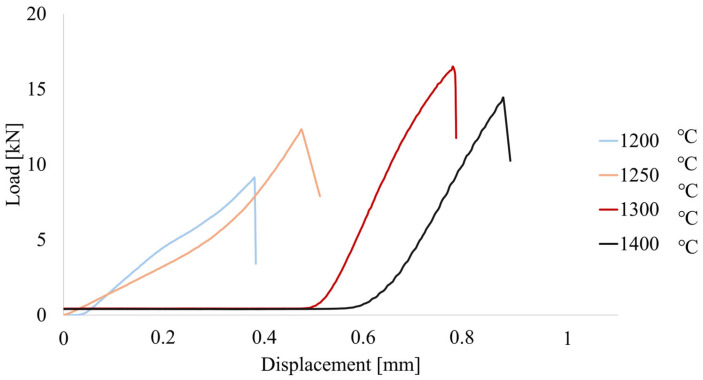
Load-displacement curve for composite samples with 2.5% vol. metal phase content for Al_2_O_3_-Cu systems formed by the PPS method. The vertical axis represents applied load [kN], and the horizontal axis represents piston displacement [mm].

Figure 6 presents the load displacement curves obtained during uniaxial compression tests of Al_2_O_3_-Cu composites fabricated at different sintering temperatures (1200 °C, 1250 °C, 1300 °C, and 1400 °C). All specimens exhibit a nonlinear increase in load with increasing piston displacement, followed by a sharp drop in load at the peak, indicating catastrophic failure driven by unstable crack propagation. Such a response is typical of alumina-based composites, in which the brittle ceramic matrix dominates the final fracture event, despite the presence of a metallic phase. A strong dependence of compressive resistance on processing temperature is observed. The peak loads increase markedly from approximately 9.2 kN at 1200 °C to 12.3 kN at 1250 °C, reaching a maximum of about 16.5 kN for specimens sintered at 1300 °C. A further increase in temperature to 1400 °C results in a decrease of the peak load to about 14.4 kN, although the displacement at failure continues to increase. This trend indicates that 1300 °C is the optimal sintering temperature for maximizing compressive strength in the investigated Al_2_O_3_-Cu system. The improvement in strength from 1200 °C to 1300 °C can be attributed to enhanced densification, improved interparticle bonding, and a reduction in critical defects such as open porosity, which delays the initiation of macroscopic cracking. At 1400 °C, the observed reduction in peak load suggests that excessive thermal exposure may lead to microstructural coarsening and redistribution of the copper phase, thereby reducing the efficiency of load transfer across the ceramic matrix and weakening the composite at peak stress. The shapes of the load-displacement curves provide insight into the damage mechanisms operating at different temperatures. Specimens sintered at 1200 °C and 1250 °C fail at relatively small displacements, and the abrupt post-peak load drop indicates that failure is controlled by the rapid formation of a dominant crack, likely initiated at residual pores or weak ceramic-metal interfaces. This behavior is consistent with insufficient consolidation and higher defect sensitivity at lower sintering temperatures.

In contrast, specimens processed at 1300 °C and 1400 °C sustain significantly higher loads and deform to larger displacements before failure. The extended nonlinear region prior to the peak load suggests the accumulation of stable microcracks and progressive interfacial debonding, possibly accompanied by localized plastic deformation of the copper phase. Such mechanisms delay catastrophic failure and increase the energy absorbed before collapse. Nevertheless, the final fracture remains unstable for all samples, as evidenced by the sudden loss of load-bearing capacity, indicating that once a critical crack network forms, the composite fails abruptly. The peak loads obtained in this study are in good agreement with previously reported compression results for Al_2_O_3_-Cu-based composites. In particular, the maximum load of about 16.5 kN for the 1300 °C series is slightly higher than the 13–15 kN reported for Al_2_O_3_-Cu composites sintered at 1400 °C in a reducing atmosphere in recent literature [56]. It is comparable to the lower end of the reported values for strengthened ternary systems, such as Al_2_O_3_-Cu-Ni composites (about 15.6 kN at low metallic content) [57]. The decrease in strength observed for the 1400 °C series in the present work is consistent with literature reports indicating that excessive sintering temperatures may deteriorate mechanical performance due to microstructural coarsening and interfacial degradation.

**Figure 7:**
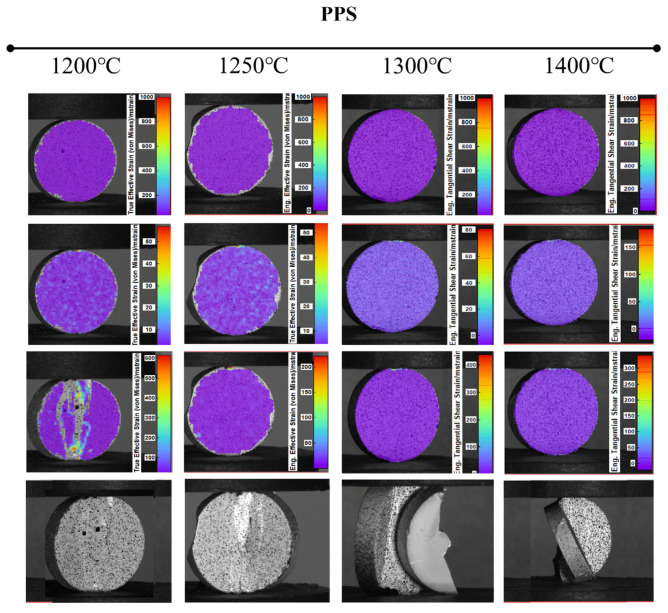
Full-field strain distribution obtained by Digital Image Correlation (DIC) during compression of Al_2_O_3_-Cu composites (2.5 vol.% Cu) sintered at 1200 °C, 1250 °C, 1300 °C, and 1400 °C. Color maps represent principal strain magnitude at the onset of failure.

The distribution of deformations during compression testing of the samples is shown in Figure 7. It was found that the shape sintered at 1200°C exhibited very low strength and a narrow elastic range. It fractured in multiple places along the direction of the load. It was characterized by a uniform but brittle structure. The sample made at 1250°C was also brittle. Under monotonic loading, numerous cracks formed along the vertical axis. The sintered material exhibited low cohesion, rendering it unsuitable for compressive loads. Fittings sintered at higher temperatures (1300°C, 1400°C) had a uniform, consistent character. They were also characterized by high brittleness and did not deform. In both sintered products, the destruction was dynamic, and a core remained between the punches along the vertical axis, which continued to transfer the load.

**Figure 8:**
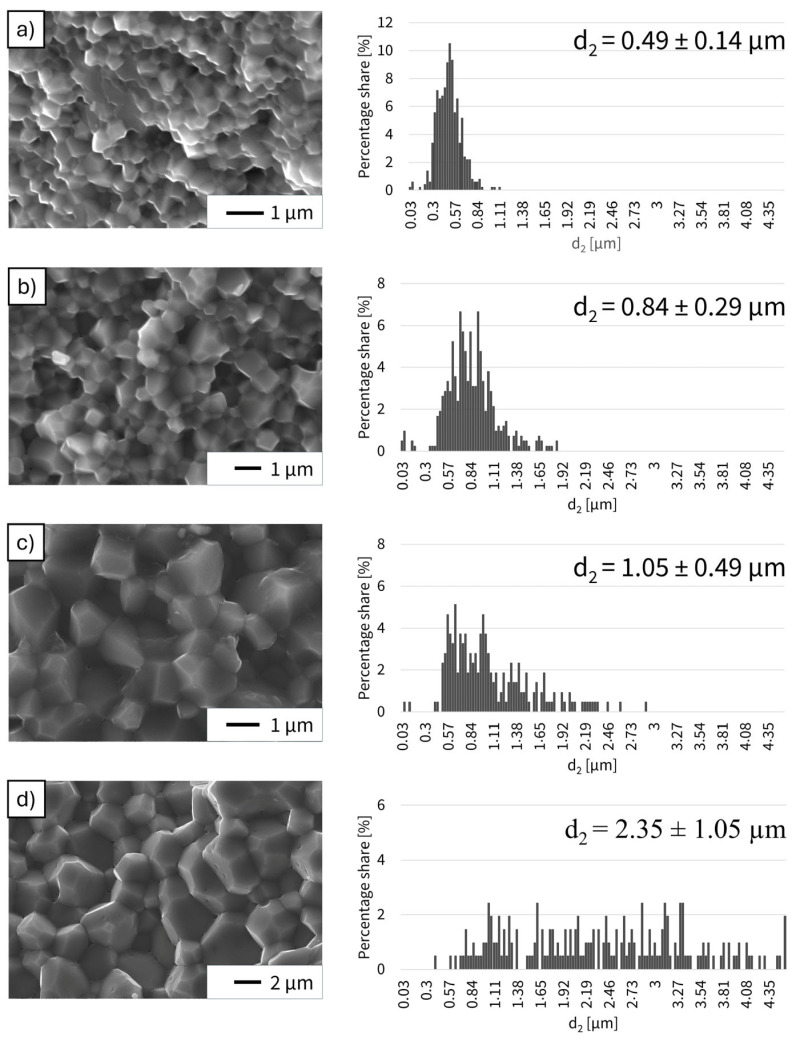
Fracture surface SEM micrographs and corresponding grain size distributions of Al_2_O_3_ in Al_2_O_3_-Cu composites (2.5 vol.% Cu) sintered by PPS at: (**a**) 1200 °C, (**b**) 1250 °C, (**c**) 1300 °C, (**d**) 1400 °C. SEM images (secondary electron mode) reveal intergranular fracture morphology and progressive alumina grain growth with increasing temperature. Histograms present the distribution of equivalent grain diameter (d_2_), determined via stereological analysis.

In samples produced using the PPS method, the analysis revealed a correlation between the sintering temperature and the average equivalent diameter. The d_2_ value in the analyzed sintered samples increased with increasing process temperature (Figure 8). The smallest grain growth was observed in the case of a melded part produced at 1200 °C, for which the d_2_ value was 0.49 ± 0.14 µm. For sintered materials formed at 1250 °C, the d_2_ value was 0.84 ± 0.25 µm, which was closest to the value obtained for the conventionally produced sample from the same system. As the process temperature increased, the Al_2_O_3_ particles continued to grow. The d_2_ value for the sample sintered at 1300 °C was 1.05 ± 0.49 µm, while for the shape sintered at 1400 °C it reached 2.35 ± 1.05 µm. When analyzing the results for this system, it can be seen that the sample with the maximum d_2_ value had an Al_2_O_3_ matrix particle size approximately 5 times larger than that in the melded part with the smallest average d_2_ value. In addition, with increasing temperature, apart from the aforementioned growth of the matrix grain, an increasing diversity of ceramic particles in terms of size was also observed in the manufactured composites. Fracture observations of these samples revealed significantly smaller pores at the grain boundaries than in the conventionally manufactured fitting. The number of pores decreased with increasing temperature and progressive growth of the matrix grain. The propagating cracks were intergranular.

Unlike our previous PPS studies on intermetallic-containing systems (e.g., NiAl-Al_2_O_3_) [24,33], the present work investigates a more demanding binary Al_2_O_3_-Cu composite in which copper melts well below the alumina sintering temperature and exhibits poor wettability with the ceramic matrix. The novelty of this study lies in demonstrating that Pulse Plasma Sintering enables effective mechanical immobilization of molten copper at only 2.5 vol.%, well below the percolation threshold, thereby isolating fundamental liquid-phase confinement mechanisms without network formation effects. Furthermore, this work establishes a clear temperature-dependent trade-off between matrix strengthening and metal-assisted toughening, identifying distinct processing windows for maximum compressive strength (1300 °C) and maximum fracture toughness (1400 °C). These results advance previous PPS research by providing mechanistic insight into copper immobilization and quantitatively linking temperature-driven microstructural evolution to competing mechanical responses in a low-metal-content ceramic–metal system.

## 4. Conclusions

The present study examines the fabrication, microstructural evolution, and mechanical performance of Al_2_O_3_-Cu ceramic-metal composites containing 2.5 vol.% copper, produced by Pulse Plasma Sintering (PPS). The primary objective was to evaluate the influence of sintering temperature, in the range of 1200–1400 °C, on densification behavior, phase stability, copper distribution, grain growth of the alumina matrix, and resulting mechanical properties, with particular attention paid to overcoming the well-known challenges associated with liquid copper migration during alumina sintering.

Composite consolidation by PPS proved highly effective across the entire temperature range investigated. All samples achieved very high relative densities, exceeding 99% of the theoretical value, with relative densities of 99.27 ± 0.20% at 1200 °C, 99.41 ± 0.65% at 1250 °C, 99.17 ± 0.47% at 1300 °C, and 99.43 ± 0.53% at 1400 °C. Open porosity and water absorption remained negligible (≤0.05% and approximately 0.01%, respectively), demonstrating that the combination of rapid heating, short dwell time (3 min), and applied uniaxial pressure inherent to PPS enables efficient pore elimination while suppressing copper leakage, despite copper being in the liquid state during processing.

X-ray diffraction analysis confirmed that all composites consisted exclusively of Al_2_O_3_ and metallic Cu, regardless of sintering temperature. No secondary reaction products or interfacial phases were detected, indicating excellent phase stability of the Al_2_O_3_-Cu system under pulsed plasma conditions and confirming that PPS does not promote undesirable chemical interactions between the ceramic matrix and the metallic phase.

Microstructural observations revealed irregularly shaped copper particles embedded in the alumina matrix, as well as regions where copper was finely dispersed between Al_2_O_3_ grains. The proportion of these dispersed metallic regions decreased systematically with increasing sintering temperature. This effect was attributed to accelerated alumina densification at higher temperatures, which led to earlier physical immobilization of liquid copper between rapidly bonding Al_2_O_3_ particles. At 1400 °C, the metallic phase distribution was notably more homogeneous, with a reduced presence of dispersed copper areas and an absence of large agglomerates or metal-depleted zones. These observations provide direct evidence that PPS promotes effective mechanical trapping of copper within the ceramic skeleton, even in the absence of favourable wettability.

Grain growth of the alumina matrix exhibited a strong dependence on sintering temperature. The average equivalent grain diameter (d_2_) increased from 0.49 ± 0.14 µm at 1200 °C to 0.84 ± 0.25 µm at 1250 °C, 1.05 ± 0.49 µm at 1300 °C, and reached 2.35 ± 1.05 µm at 1400 °C, corresponding to an almost fivefold increase across the studied temperature range. Higher temperatures also led to a broader grain-size distribution, reflecting enhanced diffusion and coarsening during sintering.

The evolution of microstructure was directly reflected in the mechanical behavior of the composites. Vickers hardness decreased monotonically with increasing sintering temperature, from 19.5 ± 2.8 GPa at 1200 °C to 18.1 ± 1.3 GPa at 1250 °C, 13.3 ± 2.0 GPa at 1300 °C, and 12.2 ± 1.6 GPa at 1400 °C. This trend is consistent with progressive alumina grain growth and the reduced constraint imposed by the ceramic matrix on the metallic phase. In contrast, fracture toughness increased with increasing temperature, reaching a maximum of 5.42 ± 0.65 MPa·m^0.5^ at 1400 °C, compared with 4.38 ± 0.51 MPa·m^0.5^ at 1200 °C. The enhanced toughness at higher temperatures is attributed to improved interfacial bonding, more homogeneous copper distribution, and increased contributions from crack deflection and localized plastic deformation of the metallic phase.

Compressive strength strongly depends on sintering temperature: peak load rises from ~9.2 kN (1200 °C) to a maximum of ~16.5 kN at 1300 °C, then decreases to ~14.4 kN at 1400 °C. The strength improvement up to 1300 °C is attributed to enhanced densification, better interparticle bonding, and reduced porosity, whereas the decline at 1400 °C likely results from microstructural coarsening and copper phase redistribution. Specimens sintered at lower temperatures fail at smaller displacements due to insufficient consolidation and higher defect sensitivity, indicating that 1300 °C is the optimal sintering temperature for maximizing compressive performance in this system.

This work demonstrates that Pulse Plasma Sintering is a highly effective technique for fabricating dense Al_2_O_3_-Cu composites with low metallic phase content. The study provides new insight into the mechanisms of copper immobilization under pulsed plasma conditions and establishes clear structure-property relationships linking sintering temperature to microstructural evolution and mechanical performance. By identifying distinct processing windows for optimizing strength and fracture resistance, the results presented here contribute to a broader understanding of advanced sintering methods for ceramic-metal systems containing low-melting metallic phases and support the development of alumina-based composites with tailored multifunctional properties.

## Figures and Tables

**Table 1:** Selected physical properties of Al_2_O_3_-Cu composites with 2.5% vol. of the metallic phase were determined using Archimedes’ method.

Temperature [°C]	Relative Density [%]	Open Porosity [%]	Water Absorption [%]
1200	99.27 ± 0.20	0.02 ± 0.01	0.01 ± 0.01
1250	99.41 ± 0.65	0.05 ± 0.01	0.01 ± 0.01
1300	99.17 ± 0.47	0.02 ± 0.01	0.01 ± 0.01
1400	99.43 ± 0.53	0.01 ± 0.01	0.01 ± 0.01

±standard deviations correspond to the statistical dispersion of individual measurements.

## Data Availability

The original contributions presented in this study are included in the article. Further inquiries can be directed to the corresponding author.
